# Mitochondrial genomics reveals the evolutionary history of the porpoises (Phocoenidae) across the speciation continuum

**DOI:** 10.1038/s41598-020-71603-9

**Published:** 2020-09-16

**Authors:** Yacine Ben Chehida, Julie Thumloup, Cassie Schumacher, Timothy Harkins, Alex Aguilar, Asunción Borrell, Marisa Ferreira, Lorenzo Rojas-Bracho, Kelly M. Robertson, Barbara L. Taylor, Gísli A. Víkingsson, Arthur Weyna, Jonathan Romiguier, Phillip A. Morin, Michael C. Fontaine

**Affiliations:** 1 Groningen Institute for Evolutionary Life Sciences (GELIFES), University of Groningen, PO Box 11103 CC, Groningen, The Netherlands; 2 Swift Biosciences, 674 S. Wagner Rd., Suite 100, Ann Arbor, MI 48103 USA; 3 IRBIO and Department of Evolutive Biology, Ecology and Environmental Sciences, Faculty of Biology, University of Barcelona, Diagonal 643, 08071 Barcelona, Spain; 4 MATB-Sociedade Portuguesa de Vida Selvagem, Estação de Campo de Quiaios, Apartado EC Quiaios, 3080-530 Figueira da Foz, Portugal; 5 CPRAM-Ecomare, Estrada Do Porto de Pesca Costeira, 3830-565 Gafanha da Nazaré, Portugal; 6 Comisión Nacional de Áreas Naturales Protegidas (CONANP), C/o Centro de Investigación Científica y de Educación Superior de Ensenada, Carretera Ensenada-Tijuana 3918, Fraccionamiento Zona Playitas, 22860 Ensenada, BC Mexico; 7 Southwest Fisheries Science Center, National Marine Fisheries Service, NOAA, 8901 La Jolla Shores Dr., La Jolla, CA 92037 USA; 8 Marine and Freshwater Research Institute, Fornubúðum 5, 220 Hafnarfjörður, Iceland; 9 Institut Des Sciences de L’Évolution (Université de Montpellier, CNRS UMR 5554), Montpellier, France; 10 Laboratoire MIVEGEC (Université de Montpellier, CNRS 5290, IRD 229) et Centre de Recherche en Écologie et Évolution de la Santé (CREES), Institut de Recherche Pour Le Développement (IRD), 911 Avenue Agropolis, BP 64501, 34394 Montpellier Cedex 5, France

**Keywords:** Biogeography, Conservation biology, Ecological genetics, Molecular ecology, Molecular evolution, Phylogenetics, Population genetics, Speciation, Evolutionary biology

## Abstract

Historical variation in food resources is expected to be a major driver of cetacean evolution, especially for the smallest species like porpoises. Despite major conservation issues among porpoise species (e.g., vaquita and finless), their evolutionary history remains understudied. Here, we reconstructed their evolutionary history across the speciation continuum. Phylogenetic analyses of 63 mitochondrial genomes suggest that porpoises radiated during the deep environmental changes of the Pliocene. However, all intra-specific subdivisions were shaped during the Quaternary glaciations. We observed analogous evolutionary patterns in both hemispheres associated with convergent evolution to coastal versus oceanic environments. This suggests that similar mechanisms are driving species diversification in northern (harbor and Dall’s) and southern species (spectacled and Burmeister’s). In contrast to previous studies, spectacled and Burmeister’s porpoises shared a more recent common ancestor than with the vaquita that diverged from southern species during the Pliocene. The low genetic diversity observed in the vaquita carried signatures of a very low population size since the last 5,000 years. Cryptic lineages within Dall’s, spectacled and Pacific harbor porpoises suggest a richer evolutionary history than previously suspected. These results provide a new perspective on the mechanisms driving diversification in porpoises and an evolutionary framework for their conservation.

## Introduction

Most cetaceans possess a tremendous potential for dispersal in an environment that is relatively unobstructed by geographical barriers. This observation begs the question of how do populations of such highly mobile pelagic species in such an open environment split and become reproductively isolated from each other and evolve into new species. Recent micro- and macro-evolutionary studies showed that changes in environmental conditions^1–6^, development of matrilineally transmitted cultures^7^, and resource specialization^8–10^ are major drivers of population differentiation and speciation in cetacean species. Yet, the processes that link these two evolutionary timescales are still not fully understood and empirical examples are limited^1, 10^.

Several cetacean taxa display an antitropical distribution where the distribution of closely related taxa occurs on either side of the equator but are absent from the tropics^11–13^. Multiple mechanisms have been proposed to explain such a peculiar distribution^14^. In cetaceans, the predominant hypothesis implies dispersal and vicariance of temperate species enabled by oceanographic, climatic and geologic fluctuations during the Miocene, Pliocene and early Pleistocene epochs^1,12,15,16^. It has been hypothesis that during cold periods, cold adapted taxa extended their range into the tropics and possibly crossed the equator. In the subsequent warmer interglacial periods, these taxa would shift their range to higher latitudes. This geographic isolation in both hemispheres promoted allopatric divergence of conspecific taxa, resulting in their antitropical distribution. A closely related scenario suggests that the rise of the overall sea temperature during interglacial periods would have altered the wind's direction and upwelling strength, leading to a redistribution of feeding areas for cetaceans toward higher latitudes, which in turn promoted their antitropical distribution^12^. Another plausible hypothesis implies that broadly distributed species, such as several cetacean species, were outperformed in the tropics by more competitive species^14^. A combination of these different mechanisms is also possible.

**Figure 1:**
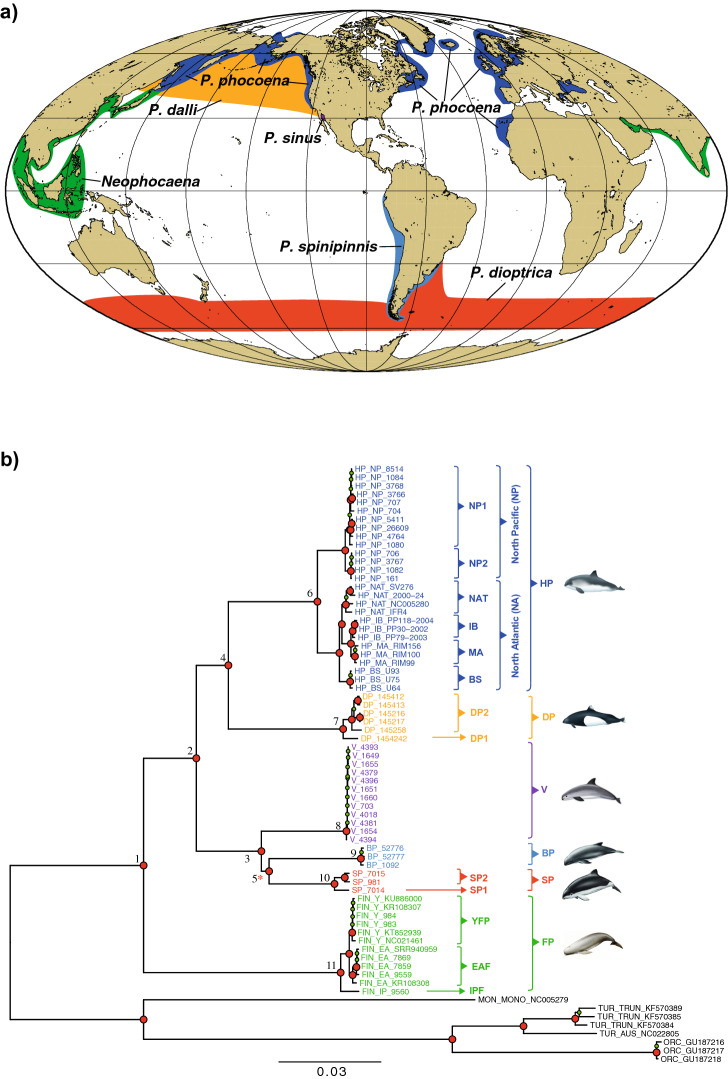
(**a**) Geographical range of each species in the Phocoenidae family. Map generated using ArcGIS 10.3 software using the open source data from the ETOPO1 Global Relief Model^23^ (https://www.ngdc.noaa.gov/mgg/global/) and adapted from Gaskin^18^ and Berta et al.^24^. (**b**) Maximum-likelihood mitochondrial phylogeny. The external branches and the tip labels are colored by species. The tree is rooted with eight sequences from four closely related *Odontoceti* species (in black). Numbers at the nodes are discussed in the text. The nodes indicated in red and green represent nodes with bootstrap support ≥ 90% and ≥ 70%, respectively. The red star indicates a node with a 63% bootstrap support in the Neighbor-joining tree (Fig. S1b). The lineage code includes the vaquita (*P. sinus*, V), Burmeister’s porpoise (*P. spinipinnis, *BP), spectacled porpoise (*P. dioptrica, *SP), Dall’s porpoise (*P. dalli, *DP), harbor porpoise (*P. phocoena, *HP) and finless porpoise (*N. phocaenoides *+ *N. asiaeorientalis, *FP). Some species have been also further subdivided into distinct groups: harbor porpoises are divided into North Pacific (NP) and North Atlantic (NA), and within each of these groups, further subdivisions are recognized. Four groups are recognized within NA: NAT (North Atlantic), IB (Iberian), MA (Mauritanian), BS (Black Sea). NP is divided into two subgroups: NP1 and NP2. Three sub-species or species are also recognized among the finless porpoises: Indo-Pacific (IPF), Yangtze finless (YFP) and East Asia finless (EAF). Spectacled porpoise subgroups are designated as SP1 and SP2. Dall's porpoise subgroups as DP1 and DP2. The scale bar unit is in substitution per site.

The porpoises family (Phocoenidae) displays one of the best known example of antitropical distribution^13^. Porpoises are among the smallest cetaceans and represent an interesting evolutionary lineage within the Delphinoidea, splitting from the Monodontidae during the Miocene (~ 15 Myr)^1,17^. Gaskin^18^ suggested that porpoises originated from a tropical environment and then radiated into temperate zones in both hemispheres. In contrast, based on the location of the oldest fossils, Barnes^13^ suggested that they arose in a more temperate environment of the North Pacific Ocean and subsequently colonized the southern hemisphere, the Indian and Atlantic Oceans. Porpoises consist of seven extant species that occur in both hemispheres in pelagic, coastal, and riverine habitats (Fig. 1a). The family includes primarily cold-water tolerant species, but two sister species of finless porpoises (*Neophocoena phocaenoides, N. asiaeorientalis*)^19^ inhabit the tropical waters of the Indo-Pacific preferring mangrove zones. They are also found in the Yellow Sea, East China Sea, Sea of Japan and in estuaries and large river systems of the Indus, Ganges, and Yangtze rivers. The long pectoral fins of these porpoises represent a potential adaptation to warm water^20^. The remaining species are considered cold-water tolerant. They are antitropically distributed and the short body appendices of most of these species are believed to represent an adaptation that limits thermal exchanges in colder environment^20^. In the Northern Hemisphere, the harbor porpoise (*Phocoena phocoena*) inhabits the coastal waters of the North Pacific and North Atlantic, while the Dall’s porpoise (*Phocoenoides dalli*) occupies the oceanic waters of the North Pacific. The large heart and high blood-oxygen of the Dall’s porpoises suggest that this species is adapted to deep diving and so probably to the oceanic environment^21^. This neritic-oceanic habitat segregation is mirrored in the southern hemisphere with the Burmeister’s porpoise (*Phocoena spinipinnis*), occupying the coastal waters of South America and the poorly known spectacled porpoise (*Phocoena dioptrica*) occupying the circum-Antarctic oceanic waters. The vaquita departs from the other species with an extremely localized geographical distribution in the upper Gulf of California and is now critically endangered^22^.

With the exception of vaquitas, all species of porpoises exhibit a relatively broad distribution range that appear fairly continuous at a global scale. Nevertheless, despite having the ability to disperse over vast distances in an open environment, many include distinct sub-species, ecotypes, or morphs. For example, the finless porpoises not only include two recognized species, but also an incipient species within the Yangtze River^19,25^; the harbor porpoise also displays a disjunct distribution with three sub-species officially recognized and an additional one suggested by Fontaine et al*.*^8^; at least two forms of Dall’s porpoise have been described^26^; and the Burmeister's porpoise also shows evidence of deep population structure^27^. Many of these subgroups show specific ecological^28^, physiological^19^ and morphological^29^ adaptations to their respective environments. For instance, Zhou et al*.*^19^ identified several genes under selection in the Yangtze finless porpoise associated with the renal function and urea cycle, reflecting adaptations to the freshwater environment. Likewise, morphological, stomach content and stable isotopes differences exclusive to Mauritanian and Iberian harbor porpoises are likely adaptations to the upwelling related environment^30^. Such intraspecific subdivisions, also observed in killer whales (*Orcinus orca*)^10^ and bottlenose dolphins (*Tursiops truncatus*)^31^, illustrate the evolutionary processes in action, and can, in some cases, eventually lead to new species. Porpoises are thus an interesting model to investigate the evolutionary processes at both micro- and macroevolutionary time scale to better understand present and historical mechanisms driving population structure, adaptation to different niches, and speciation.

From a conservation perspective, the coastal habitat of many porpoise species largely overlaps with areas where human activities are the most intense. These have dramatic impacts on natural populations. For example, the vaquita lost 90% of its population between 2011 and 2016 leaving about 30 individuals in 2017^32^, and less than 19 in 2019^33^. This species is on the brink of extinction and currently represents the most endangered marine mammal. Finless porpoises also face major conservation issues, especially the lineage within the Yangtze River (*N. a. asiaeorientalis*) in China, also critically endangered due to human activities^34,35^. Similarly, several populations of harbor porpoises are highly threatened^36,37^. Little information about the spectacled and Burmeister’s porpoises is available. While anthropogenic activities are an undeniable driver of the current threats to biodiversity, the evolutionary context can be also informative when assessing their vulnerability^38^. For example, knowledge on population or species evolutionary history is useful to characterize population dynamics, identify evolutionary significant units relevant for conservation, recent or historical split related to environmental variation, evolutionary or demographic trends, and evolutionary processes that could explain, enhance, or mitigate the current threats experienced by a species^39,40^.

To date, porpoise evolutionary history and biogeography remains contentious and superficial^41^. Previous phylogenetic studies led to incongruent results, as there are disagreements regarding some of their relationships, in particular about the position of the vaquita, Dall's, Burmeister's and spectacled porpoises^13,41,42^. So far, molecular phylogenetic relationships among porpoises have been estimated using short sequences of the *D-loop* and cytochrome *b*^17,42^. However, the *D-loop* can be impacted by high levels of homoplasy that blurs the resolution of the tree^43^ and the *Cyt-b* may have limited power to differentiate closely related taxa^44^.

In this study, we sequenced and assembled the whole mitogenome from all extant porpoise species, including most of the known lineages within species to resolve their phylogenetic relationships and reconstruct their evolutionary history. More specifically, (1) we assessed the phylogenetic and phylogeographic history of the porpoise family based on the whole mitogenomes including the timing and tempo of evolution among lineages; (2) we assessed the genetic diversity among species and lineages and (3) reconstructed the demographic history for some lineages for which the sample size was suitable. (4) We placed the evolutionary profile drawn for each lineage and species into the framework of past environmental changes to extend our understanding of porpoise biogeography. Finally, (5) we interpreted the IUCN conservation status of each taxa in the light of their evolutionary history.

## Material and methods

### Taxon sampling and data collection

Porpoise tissue samples from 56 live-biopsies, bycaught, or stranded animals (Table 1 and Table S1) were stored in salt-saturated 20% DMSO or 95% Ethanol and stored at − 20 °C until analyses. All samples were collected under appropriate Marine Mammal Protection Act permits within the US, or appropriate permits elsewhere, following the relevant guidelines and regulations, and transferred internationally under CITES permit. Genomic DNA was extracted from tissues using the *PureGene* or *DNeasy* Tissue kits (Qiagen), following the manufacturer’s recommendations. DNA quality and quantity were assessed on an agarose gel stained with ethidium bromide, as well as using a Qubit-v3 fluorometer. Genomic libraries for 44 porpoise samples including three spectacled porpoises, three Burmeister's porpoises, 12 vaquita, six Dall's porpoises, three East Asian finless porpoises, two Yangtze finless, one Indo-Pacific finless and 14 North Pacific harbor porpoises. Libraries were prepared by Swift Biosciences Inc. using either the Swift Biosciences Accel-NGS double-strand 2S (harbor porpoises) or single-strand 1S Plus DNA Library Kit (all other species), following the user protocol and targeting an insert-size of ~ 350 base-pairs (bps). The libraries were indexed and pooled in groups of 2–12 for paired-end 100 bps sequencing in five batches on an Illumina MiSeq sequencer at Swift Biosciences. Additional libraries for 12 samples of North Atlantic harbor porpoises were prepared at BGI Inc. using their proprietary protocol, indexed and pooled for paired-end 150 bps sequencing on one lane of HiSeq-4000 at BGI Inc. The total sequencing effort produced reads for 56 individuals (Table S2). Previously published reads from one additional finless porpoise sequenced with a Hiseq-2000 were retrieved from NCBI (Table S2). For this individual, we down-sampled the raw FASTQ files to extract 0.5% of the total reads and used the 5,214,672 first reads to assemble the mitogenome. The subsequent data cleaning and mitogenome assemblies were thus performed for a total of 57 individuals.

**Table 1:** Taxon sample size (n) and descriptive statistics for the shotgun sequencing and mitochondrial assembly per species and mitochondrial lineage.

Lineage	n	R_*b*_	R_*a*_	Depth	Assembly size	% GC
Harbor porpoise (HP)	27	30.8 ± 28.3	28.5 ± 25.9	1,323.9 ± 1,800.0	16,383.8 ± 0.8	40.6 ± 0.1
North Pacific 1 (NP1)	10	5.1 ± 1.8	5.0 ± 1.7	99.6 ± 92.6	16,384.3 ± 0.5	40.7 ± 0.0
North Pacific 2 (NP2)	4	5.9 ± 1.3	5.6 ± 1.2	97.2 ± 119.8	16,384.0 ± 0.0	40.7 ± 0.0
North Atlantic (NAT)	4	56.9 ± 3.4	52.2 ± 2.9	1656.3 ± 1,058.9	16,383.3 ± 0.6	40.6 ± 0.0
Mauritania (MA)	3	59.8 ± 5.4	54.9 ± 5.2	1,431.0 ± 648.1	16,384.0 ± 0.0	40.6 ± 0.0
Iberia (IB)	3	65.2 ± 8.2	60.5 ± 7.6	3,169.7 ± 1882.6	16,384.0 ± 0.0	40.5 ± 0.0
Black Sea (BS)	3	60.0 ± 2.2	54.8 ± 1.3	4,755.3 ± 1,376.9	16,382.0 ± 0.0	40.6 ± 0.0
Dall’s porpoise (DP)	6	2.8 ± 0.9	2.7 ± 0.9	49.5 ± 14.5	16,367.5 ± 0.8	40.5 ± 0.1
Dall's porpoise 1 (DP1)	1	4.5 ± 0.0	4.4 ± 0.0	74.0 ± 0.0	16,369.0 ± 0.0	40.6 ± 0.0
Dall's porpoise 2 (DP2)	5	2.5 ± 0.5	2.4 ± 0.5	44.6 ± 9.0	16,367.2 ± 0.4	40.5 ± 0.0
Vaquita (V)	12	5.8 ± 5.7	5.7 ± 5.5	113.3 ± 108.6	16,370.0 ± 0.0	39.8 ± 0.0
Burmeister’s porpoise (BP)	3	2.3 ± 0.3	2.3 ± 0.3	125.0 ± 121.2	16,378.7 ± 8.1	40.1 ± 0.0
Spectacled porpoise (SP)	3	2.4 ± 0.2	2.3 ± 0.2	55.0 ± 30.5	16,371.0 ± 0.0	39.7 ± 0.1
Spectacled porpoise 1 (SP1)	1	2.2 ± 0.0	2.1 ± 0.0	41.0 ± 0.0	16,371.0 ± 0.0	39.6 ± 0.0
Spectacled porpoise 2 (SP2)	2	2.5 ± 0.0	2.4 ± 0.0	62.0 ± 39.6	16,371.0 ± 0.0	39.7 ± 0.1
Finless porpoises (FP)	12	2.7 ± 1.1	2.6 ± 1.1	92.9 ± 74.9	16,383.1 ± 5.8	40.8 ± 0.0
Indo-Pacific (IPF)	1	2.3 ± 0.0	2.2 ± 0.0	77.0 ± 0.0	16,385.0 ± 0.0	40.8 ± 0.0
East Asian (EAF)	5	3.0 ± 1.5	2.9 ± 1.4	53.2 ± 22.8	16,381.2 ± 7.5	40.0 ± 0.0
Yangtze (YF)	6	2.3 ± 0.3	2.2 ± 0.3	180.0 ± 101.8	16,386.0 ± 0.0	40.8 ± 0.0

The statistics include the total number of reads before and after filtering (R_*b*_ and R_*a*_), the sequencing coverage depth, the size of the mitochondrial assembly (in base-pairs), and the GC content in percent (%GC). The mean value and the standard deviation are shown.

### Data cleaning

The quality of the reads was first evaluated for each sample using *FastQC* v.0.11.5^45^. *Trimmomatic* v.0.36^46^ was used to remove low quality regions, overrepresented sequences and Illumina adapters. Different filters were applied according to the type of Illumina platform used (see Text S1 for details). Only mated reads that passed *Trimmomatic* quality filters were used for the subsequent analyses.

### Mitogenome assembly

Porpoises mitogenome assemblies were reconstructed using two different approaches. First, we used *Geneious* v.8.1.8^47^ to perform a direct read mapping to the reference mitogenome of the harbor porpoise (accession number AJ554063^48^). We used default settings except the minimum mapping quality set to 20 and the number of iterations set to 5. This step was followed by a reconstruction of the consensus sequences (Table S2). The second approach implemented in *MITOBIM*^49^ is a hybrid approach combining a baiting and iterative elongation procedure to perform a de-novo assembly of the mitogenome (see details in Text S2). We visually compared the assemblies provided by the two methods in *Geneious* to assess and resolve inconsistencies (Text S2 and Table S2).

In addition to the 57 assembled individuals, we retrieved six porpoise mitogenomes from Genbank (Table S2)*.* We also added eight complete mitogenomes from four outgroup species: one narwhal (*Monodon monoceros*)^48^*,* three bottlenose dolphins^6^*,* one Burrunan dolphin (*Tursiops australis*)^6^ and three orcas^50^.

### Sequences alignments

We performed the alignment of the 71 mitogenomes with *MUSCLE*^51^ using default settings in *Geneious*. A highly repetitive region of 226 bps in the *D-loop* was excluded from the final alignment (from position 15,508 to 15,734) because it was poorly assembled, and included many gaps and ambiguities. We manually annotated the protein-coding genes (CDS), tRNA and rRNA of the final alignment based on a published harbor porpoise mitogenome^48^. Contrary to the remaining CDSs, *ND6* is transcribed from the opposite strand^52^. Therefore, to assign the codon positions in this gene, we wrote a custom script to reverse complement *ND6* in the inputs of all the analyses that separates coding and non-coding regions of the mitogenomes. This led to a 17 bps longer alignment due to the overlapping position of *ND5* and *ND6*.

### Phylogenetic relationships

We estimated the phylogenetic relationships among the assembled mtDNA sequences using three approaches: a maximum-likelihood method (ML) in *PHYML* v3.0^53^; a distance based method using the Neighbour-Joining algorithm (NJ) in *Geneious*; and an unconstrained branch length Bayesian phylogenetic tree (BI) in *MrBayes* v3.2.6^54^. We used the Bayesian information criterion (*BIC*) to select the substitution model that best fits the data in *jModelTest2* v2.1.10^55^. The best substitution model and parameters were used in the ML, NJ and BI approaches. For the ML approach, we fixed the proportion of invariable sites and the gamma distribution parameters to the values estimated by *jModelTest2.* The robustness of the ML and NJ tree at each node was assessed using 10,000 bootstrap replicates. For the Bayesian inference, a total of 1 × 10^6^ MCMC iterations was run after a burn-in of 1 × 10^5^ steps, recording values with a thinning of 2,000. We performed ten independent replicates and checked the consistency of the results. Runs were considered to have properly explored the parameter space if the effective sample sizes (ESS) for each parameter was greater than 200 and by visually inspecting the trace plot for each parameter using *Tracer* v1.6^56^. We assessed the statistical robustness and the reliability of the Bayesian tree topology using the posterior probability at each node.

Finally, the four phylogenetic trees were rooted with the eight outgroup sequences and plotted using the R package *ggtree* v1.4^57^.

### Divergence time estimate

We estimated the divergence time of the different lineages using a time-calibrated Bayesian phylogenetic analysis implemented in *BEAST* v2.4.3^58^. We assumed two calibration points in the tree: (1) the split between the Monodontidae and Phocoenidae, node calibrated with a lognormal mean age at 2.74 Myr^17^ (sd = 0.15) as a prior and (2) the split between the Pacific and Atlantic harbor porpoise lineages, node calibrated with a uniform distribution between 0.7 and 0.9 Myr as a prior^2^.

Divergence times were estimated using a relaxed log-normal molecular clock model for which we set the parameters *ucldMean* and *ucldStdev* to exponential distributions with a mean of 1 and 0.3337, respectively. We used a Yule speciation tree model and fixed the mean of the speciation rate parameter to 1. The *BIC* was used in *jModelTest2* to identify the substitution model best fitting to the data, using the empirical base frequencies. We assumed a substitution rate of 5 × 10^–8^ substitutions per-site and per-year. This mutation rate was estimated by Nabholz et al*.*^59^ for cetacean mitogenomes and was previously used on harbor porpoise^8,60^. A total of 1.2 × 10^9^ MCMC iterations were run after a burn-in length of 1.2 × 10^8^ iterations, with a thinning of 5,000 iterations. We performed eight independent replicates and checked for the consistency of the results among replicates. A run was considered as having converged if the *ESS* values were greater than 200, and if they produced consistent trace plots using *Tracer* v1.6. Subsequently, we combined all runs together after a burn-in of 98% using *LogCombiner*^58^. The best supported tree topology was the one with the highest product of clade posterior probabilities across all nodes (maximum clade credibility tree), estimated using *TreeAnnotator*^58^. We also calculated the 95% highest posterior density for the node ages using *TreeAnnotator*. The final chronogram was rooted with the eight outgroups sequences and plotted using *FigTree* v.1.4.3^61^.

### Genetic diversity within species and sub-species

We subdivided each species into their distinct lineages in order to compare their genetic diversity at the different taxonomic level. Specifically, we divided the harbor porpoise into five subgroups, North Pacific (*P. p. vomerina*), Black Sea (*P. p. relicta*), Mauritanian—Iberian (*P.p. meridionalis*) and North Atlantic (*P. p. phocoena*) in accordance with the subdivisions proposed for this species in the literature^30^. Finless porpoise was split into Indo-Pacific finless (*N. phocaenoides;* IPF), East Asian finless (*N. a. sunameri*; EAF) and Yangtze finless porpoises (*N. a. asiaeorientalis*; YFP). For simplicity, we refer here to finless porpoises as a single group of species and IPF, EAF and YFP as the distinct lineages throughout this paper. Additionally, we subdivided the other groups into lineages that were as divergent or more divergent than the sub-species that were described in the literature. This includes splitting the North Pacific harbor porpoises into NP1 and NP2, Dall's porpoises into DP1 and DP2 and spectacled porpoises into SP1 and SP2 to reflect the deep divergence observed in the phylogenetic tree within these three lineages (Fig. 1b and Fig. S1).

For each species and subgroup, several statistics capturing different aspects of the genetic diversity were calculated for different partitions of the mitogenome, including the whole mitogenomes, the non-coding regions (i.e. inter-gene regions and *D-loop*) and the 13 protein coding genes (e.g. CDS) excluding tRNAs and rRNA. The number of polymorphic sites, nucleotide diversity (*π*), number of singletons, number of shared polymorphisms, number of haplotypes, haplotype diversity and Watterson estimator of *θ* were calculated. For CDSs, we also estimated the number of synonymous (*#Syn*) and non-synonymous mutations (*#NSyn*), *π* based on synonymous (*π*_*S*_) and non-synonymous mutations (*π*_*N*_), and the ratio *π*_*N*_*/π*_*S*_. All these statistics were computed in *DnaSP* v.5.10.01^62^. Since we only have a unique sample for IPF, DP1 and SP1 we did not estimate these statistics for these lineages.

Differences in sample sizes can influence some of these statistics. As our sample size ranged from three to 26 individuals per group, we used a rarefaction technique^63^ to account for the differences in sample size. We assumed a sample size of three individuals in order to compare the genetic diversity among lineages that have different sample sizes. For each lineage, we randomly subsampled 2,500 times a pool of three sequences and estimated the median, mean and 95% confidence interval for *π*.

### Test for selective neutrality

We tested for evidence of natural selection acting on the mitogenomes using a McDonald–Kreitman test^64^ (MK-tests). This test infers deviation from the neutral theory by comparing the ratio of divergence between species (*d*_*N*_*/d*_*S*_) versus polymorphism within species (*π*_*N*_*/π*_*S*_) at synonymous (silent) and non-synonymous (amino acid-changing) sites in protein coding regions using the so-called neutrality index (*NI*). *NI* is 1 when evolution is neutral, greater than 1 under purifying selection, and less than 1 in the case of positive selection. MK-tests were conducted on the 13 CDS regions of the mitogenome using *DnaSP*. We conducted this test in two different ways: first comparing all the interspecific lineages to a same outgroup, the killer whale for which multiple mitogenome sequences were available, and second comparing all interspecific lineages to each other in order to assess how the MK-tests were affected by the outgroup choice. The significance of the *NI* values was evaluated using a G-tests in *DnaSP*. Furthermore, the distribution of *NI* values for each lineage were compared among each other using a PERMANOVA with the R package *RVAideMemoire v.0.9-77*^65^. Pairwise comparisons were assessed with a permutation tests and were adjusted for multiple comparisons using the false rate discovery method^66^. The PERMANOVA and pairwise comparisons were conducted using 9,999 permutations. The neutral theory predicts that the efficacy of purifying selection increases with *Ne*^67^. Under these assumptions, *Ne* is expected to be proportional to *NI*^68,69^. To test this hypothesis, we assessed the correlation between values of *NI*s and *π* derived by rarefaction as a proxy of *Ne*. However, MK-test is also known to be impacted by demographic changes in some specific cases. For instance, an increase in *Ne* could mimic the effect of positive selection^70^ while recent reduction in *Ne* could prevent the detection of positive selection and lead to an artefactual signal of purifying selection^71^. This problem is exacerbated in species with very low *Ne* and the results of MK-tests should be interpreted accordingly.

In addition to the MK-tests, we quantified the branch-specific non-synonymous to synonymous substitution ratios (*d*_*N*_*/d*_*S*_) to infer direction and magnitude of natural selection along the phylogenetic tree. To estimate the branch-specific ratio we first counted the number of synonymous (*#S*) and non-synonymous *(#NS*) substitutions for the 13 CDSs. Then *#S* and *#NS* were mapped onto a tree using the mapping procedure of Romiguier et al*.*^72^. Next, we divided *#S* and *#N* by the number of synonymous and nonsynonymous sites to obtain an approximation of *d*_*S*_ and *d*_*N*_, respectively. More specifically, we first fitted the YN98 codon model using the *BPPML* program^73^, then we mapped the estimated *d*_*N*_/*d*_*S*_ values onto the branches of the ML tree using the program *MAPNH* of the *TESTNH* package v1.3.0^74^. Extreme *d*_*N*_/*d*_*S*_ ratio (> 3) are often due to branches with very few substitution (*d*_*N*_ or *d*_*S*_)^72^ and were discarded. We compared the distribution of *d*_*N*_/*d*_*S*_ among species (i.e., across all the branches) using a PERMANOVA. Finally, the estimated ratios were correlated with *π* values obtained by rarefaction using a Pearson's correlation tests in R^75^. To do so, we pooled the signal from each lineage as a single data point as suggested by Figuet et al*.*^76^. We considered the intraspecific and interspecific lineages, except for those where no non-synonymous substitutions were observed (ex. NP2). Within a lineage, *π* was summarized as the mean of the log_10_-transformed value of its representatives and the *d*_*N*_/*d*_*S*_ was obtained by summing the non-synonymous and synonymous substitution counts across its branches and calculating the ratio^76^.

### Inference of demographic changes

We investigated changes in effective population size (*Ne*) through time for the lineages that included a sample size ≥ 10 to conduct reliable demographic inferences. This includes the vaquitas and North Pacific NP1 harbor porpoise lineage. We first tested for deviations from neutral model expectations using three statistics indicative of historical population size changes: Tajima’s *D*^77^, Fu and Li’s D* and F*^78^ in *DnaSP*. The *p*-values were assessed using coalescent simulations in *DnaSP* using a two tailed test as described in Tajima^77^. We then reconstructed the mismatch distributions indicative of population size changes using *Arlequin* v.3.5.2.2^79^. Mismatch distributions were generated under a constant population size model and a sudden growth/decline population model^80^. This later model is based on three parameters: the population genetic diversity before the population size change (*θ*_*i*_); the population genetic diversity after the population size change (*θ*_*f*_), and the date of the change in population size in mutational units (*τ* = *2.µ.t*, where *µ* is the mutation rate per sequence and generation and *t* is the time in generations). These parameters were estimated in *Arlequin* using a generalized non-linear least-square approach. The 95% confidence interval was obtained using 10,000 parametric bootstraps^80^. Finally, we used the coalescence based Bayesian Skyline Plot (BSP)^81^ to estimate demographic changes in *Ne* back to the *T*_*MRCA*_. BSP analysis was performed in *BEAST* v2.4.3 using the empirical base frequencies and a strict molecular clock. We applied *jModelTest2* separately to both lineages to evaluate the best substitution models. We assumed a substitution rate of 5 × 10^–8^ substitutions per site and per year^59^ in order to obtain the time estimates in years. We conducted a total of ten independent runs of 10^8^ MCMC iterations following a burn-in of 1 × 10^7^ iterations, logging every 3,000 iterations. We constrained *Ne* between 0 and 150,000 individuals and between 0 and 380,000 individuals for the vaquita and the NP1 harbor porpoise lineage, respectively. This upper boundary on *Ne* was empirically set to encompass the entire marginal posterior distribution. All other parameters were kept at default values. The convergence of the analysis was assessed by checking the consistency of the results over ten independent runs. For each run, we also used *Tracer* to inspect the trace plots for each parameter to assess the mixing properties of the MCMCs and estimate the *ESS* value for each parameter. Runs were considered as having converged if they displayed good mixing properties and if the *ESS* values for all parameters were greater than 200. We discarded the first 10% of the MCMC steps as a burn-in and obtained the marginal posterior parameter distributions from the remaining steps using *Tracer*. *Ne* values were obtained by assuming a generation time of 10 years^36^. To test whether the inferred changes in *Ne* over time were significantly different from a constant population size null hypothesis, we compared the BSP of both lineages with the ‘Coalescent Constant Population’ model (CONST)^58,82^ implemented in *BEAST* v2.4.3 using Bayes Factors^83^. We thus conducted ten independent CONST runs using 10^8^ MCMC iterations after a burn-in of 10%, logging every 3,000 iterations. We assessed the proper mixing of the MCMC and ensured *ESS* were greater than 200. We then used the Path sampler package in *BEAST* v2.4.3 to compute the log of marginal likelihood (*logML*) of each run for both BSP and CONST. We set the number of steps to 100 and used 10^8^ MCMC iterations after a burn-in of 10%. Bayes Factors were computed as twice the difference between the log of the marginal likelihoods (i.e. 2[*LogML*_*BSP*_ − *LogML*_*CONST*_]) and were performed for pairwise comparisons between BSP and CONST runs. As recommended, Bayes Factors greater than 6 were considered as a strong evidence to reject the null hypothesis (i.e. CONST)^83^.

## Results

### Porpoise mitogenomes assemblies

A total of 57 mitogenomes of the seven species of porpoise (Table S1) were newly sequenced and assembled using Illumina sequencing. After read quality check, trimming, and filtering (Table 1 and Table S2), between 1,726,422 and 67,442,586 cleaned reads per sample were used to assemble the whole mitogenomes. The two methods used to assemble the mitogenomes delivered consistent assemblies with an average sequencing depth coverage ranging from 15 to 4,371X for *Geneious* and 17 to 4,360X for *MITOBIM* (Table 1 and Table S2). In total, 35 of the 57 mitogenome assemblies were strictly identical with both methods. The 22 remaining assemblies included between 1 and 4 inconsistencies which were visually inspected and resolved (Text S2 and Table S2). Augmented with the 14 previously published mitogenome sequences, the final alignment counted 71 mitogenome sequences of 16,302 bps and included less than 0.2% of missing data. The alignment was composed of 627 singletons and 3,937 parsimony informative sites defining 68 haplotypes (including the outgroup sequences). Within the 63 ingroup porpoise sequences, we observed 2,947 segregating sites, including 242 singletons and 2,705 shared polymorphisms defining 58 distinct haplotypes with a haplotype diversity of 99.6% (Table 2, Tables S3 and S4).

**Table 2:** Summary statistics describing the genetic diversity of the mitochondrial genomes among porpoise species and their distinct lineages.

	N	MD	S	Singl	Shared P	θ_W_ (%)	π (%)	SD_π_ (%)	H	H_d_ (%)	SD_HD_ (%)
**Species**											
All porpoises	63	51	2,947	242	2,705	4.28	5.35	0.23	58	99.6	0.04
Finless porpoise^a^	12	36	229	173	56	0.47	0.35	0.00	12	100.0	3.40
Burmeister's porpoise^b^	3	31	33	33	0	0.13	0.13	0.04	3	100.0	27.20
Vaquita^b^	12	32	16	13	3	0.03	0.02	0.00	8	89.4	7.80
Spectacled Porpoise^a^	3	30	145	145	0	0.59	0.59	0.21	3	100.0	27.20
Dall's Porpoise^a^	6	34	208	152	56	0.56	0.49	0.13	5	93.3	14.80
Harbor Porpoise^a^	27	37	602	158	444	0.98	1.11	0.06	26	99.7	1.10
**Harbor porpoise**											
North Atlantic^b^	4	36	102	85	17	0.34	0.33	0.07	4	100.0	3.12
Iberia^b^	3	32	31	31	0	0.12	0.12	0.04	3	100.0	27.20
Mauritania^b^	3	32	28	28	0	0.11	0.11	0.03	3	100.0	27.20
Black Sea^b^	3	34	18	18	0	0.07	0.07	0.02	3	100.0	27.20
North Pacific 1^b^	10	31	76	53	23	0.16	0.12	0.01	10	100.0	4.50
North Pacific 2^b^	4	31	6	4	2	0.02	0.02	0.00	3	83.3	4.94
**Finless porpoise**											
Yangtze^b^	6	34	33	30	3	0.09	0.07	0.01	6	100.0	0.93
East Asian^b^	5	33	58	55	3	0.17	0.15	0.04	5	100.0	16.00
**Dall's porpoise**											
Dall's porpoise 2^b^	5	33	107	61	46	0.32	0.32	0.07	4	90.0	16.10
**Spectacled porpoise**											
Spectacled porpoise 2	2	29	39	39	0	0.24	0.24	0.12	2	100.0	50.00

The statistics includes the sample size (*N*), number of sites with missing data in number of gaps (*MD*), segregating sites (*S*), singletons (*Singl*.), shared polymorphism (*Shared P.),* Watterson's theta *(θ*_*W*_), average nucleotide diversity per site (*π*) and its standard deviation (*SD*_*π*_), number of haplotypes (*H*), haplotypic diversity (*H*_*d*_) and its standard deviation (*SD*_*HD*_).

^a^Species including multiple distinct mitochondrial lineages.

^b^Species with a single mitochondrial lineage.

### Phylogenetic history of the porpoises

A Hasegawa–Kishino–Yano (HKY + G, Gamma = 0.19) model was selected as the best-fitting nucleotide substitution model. Phylogenetic inferences using a maximum-likelihood (ML) approach (Fig. 1b and Fig. S1a), a distance-based neighbor-joining method (Fig. S1b), and a Bayesian approach (Fig. S1c) all produced concordant phylogenies (i.e., similar topologies and statistical supports). All phylogenies were fully resolved with high statistical support at the inter- and intra-specific levels (bootstrap supports ≥ 93% or node posterior probability of one). One exception was the node 5 grouping the Burmeister's and spectacled porpoises in the neighbor-joining tree (Fig. S1b) as it displayed a slightly lower bootstrap support of 61% (red star in Fig. 1b and Fig. S1b), but it was fully supported by the ML and Bayesian approaches (Fig. 1b and Fig. S1).

The resulting phylogenetic reconstruction (Fig. 1b) showed that all porpoises formed a monophyletic group (node 1). The most basal divergence in the tree split the more tropical finless porpoises from the other temperate to subpolar porpoises. Then, the temperate species split into two clades (node 2) composed of two reciprocally monophyletic groups. The first is composed of the southern hemisphere species (spectacled and Burmeister's porpoises) and vaquitas (node 3). The second is composed of the porpoises from the northern hemisphere (harbor and Dall’s porpoises, node 4). In contrast with a previous phylogenetic study based on control region sequences^42^, the phylogenetic tree based on the whole mitogenome suggested that vaquitas split from a common ancestor to the spectacled and Burmeister's porpoises (node 3), and thus that the two species from the southern hemisphere are more closely related to each other (node 5) than they are to vaquitas. Finally, the mitogenome tree supported the monophyly of each recognized species (nodes 6–11).

Intraspecific subdivisions were also evident from the mitogenome phylogeny in some species, such as in the harbor and finless porpoises (Fig. 1b). In the harbor porpoises, the split between the North Atlantic and North Pacific sub-species constituted the deepest divergence of all intraspecific subdivisions across all species. Within the North Atlantic harbor porpoises, further subdivisions were also observed and corresponded to the three previously described ecotypes or sub-species^30^. These included the relict population in the Black Sea, the harbor porpoises from the upwelling waters with two closely related but distinct lineages in Iberia and Mauritania, and the continental shelf porpoises further north in the North Atlantic. Finally, within the North Pacific, two cryptic subgroups were also observed (NP1 and NP2; Fig. 1b). Among the finless porpoises, the three taxonomic groups currently recognized^19^, including IPF and the two closely related species of narrow-ridged finless porpoises, were clearly distinct from each other on the mitogenome phylogenetic tree (node 11). Finally, despite a limited sampling, Dall's (node 7) and spectacled porpoises (node 10) each also displayed distinct lineages (DP1/DP2 and SP1/SP2, respectively; Fig. 1b and Fig. S1) as divergent as those observed in the harbor and finless porpoises.

**Figure 2:**
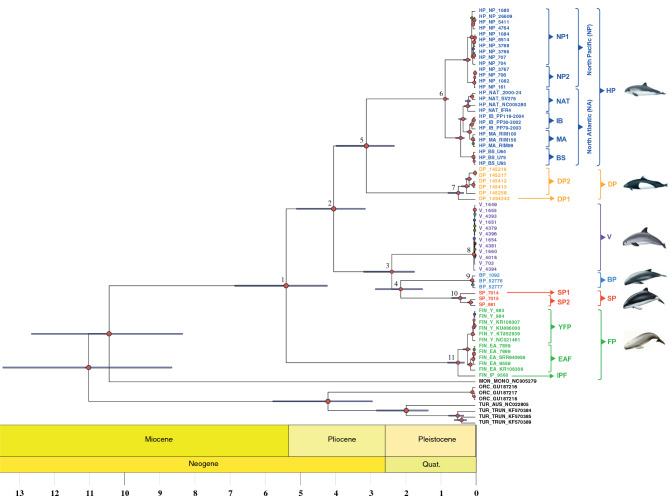
Bayesian chronogram of the porpoise family. The tree represents the maximum clade credibility tree. Red node labels indicate posterior probabilities of 1; node position indicates median node age estimates and the error bars around the node indicate the 95% highest posterior density of the estimated node age. Time is in millions of years. Numbers at the nodes are discussed in the text. The acronyms are provided in Fig. 1.

The time-calibrated Bayesian mitochondrial phylogeny (Fig. 2 and Table S5) suggested that all extant porpoises find their common ancestor ~ 5.42 M years ago (95% Highest Posterior Density, HPD, 4.24–6.89; node 1). This time corresponds to the split between the finless and the other porpoise species. Spectacled, vaquita and Burmeister's porpoises diverged from harbor and Dall's porpoise ~ 4.06 Myr ago (95% HPD, 3.15–5.12; node 2). The split between vaquitas, spectacled and Burmeister's porpoises was estimated at ~ 2.39 Myr (95% HPD, 1.74–3.19; node 3) and between spectacled and Burmeister's at ~ 2.14 Myr (95% HPD, 1.51–2.86; node 4). The Dall's and harbor porpoises split from each other ~ 3.12 Myr ago (95% HPD, 2.31–3.98; node 5). Finally, the common ancestor of the subdivisions observed within each species was dated within the last million years (nodes 6–11).

### Genetic diversity of the mitogenome

**Figure 3:**
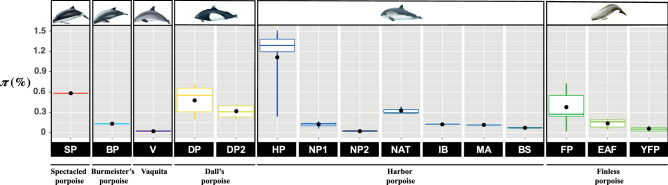
Nucleotide diversity (*π*) among species and lineages within species of porpoise. The median and mean *π* values are represented respectively by the colored line in the boxplot and the black dot. The whiskers represent the 95% confidence interval. The boxes represent the upper and lower quartile. No overlapping boxplots are significantly different. The species are represented by a pictogram on the top of the figure. The names of the distinct lineages are provided at the bottom of the plot in the black boxes. The acronyms are provided in Fig. 1 and Table 1.

Mitochondrial genetic diversity varied greatly among species and lineages within species (Table 2, Tables S3, S4 and Fig. 3). The highest values of *π* were observed in the harbor porpoises (*π* = 1.15%), followed by the spectacled (*π* = 0.60%), Dall's (*π* = 0.50%), finless (*π* = 0.35%), and Burmeister's porpoises (*π* = 0.13%), while vaquitas displayed the lowest values (*π* = 0.02%). The variation among species was strongly related to the occurrence of distinct mitochondrial lineages within species that corresponds to ecologically and genetically distinct sub-species or ecotypes (Table 2, Tables S3, S4 and Fig. 3). Once the lineages that included more than three sequences were compared to each other while accounting for the difference in sample size using a rarefaction procedure^63^ (Fig. 3), π values were more homogeneous among lineages and species, with however some variation. The most diversified lineages included DP2 in Dall's porpoise (*π* = 0.32%) and the North Atlantic (NAT) lineage in harbor porpoise (*π* = 0.33%). In contrast, the vaquita lineage (*π* = 0.02%), harbor porpoise North Pacific lineage NP2 (*π* = 0.02%) and Black Sea lineage (BS) (*π* = 0.07%), and the Yangtze finless porpoise YFP lineage (*π* = 0.06%) displayed the lowest nucleotide diversity. The other lineages displayed intermediate *π* values.

### Molecular evolution of the mitogenome

The nucleotide diversity also varied greatly along the mitogenome. It was lowest in the origin of replication, tRNA and rRNAs, intermediate in the coding regions and highest in non-coding regions (Fig. S2, Tables S3 and S4). This result indicates different levels of molecular constraints along the mitogenome.

The *π*_*N*_*/π*_*S*_ ratio in the 13 CDSs, displaying the relative proportion of non-synonymous versus synonymous nucleotide diversity, was lower than one in all the lineages. This is consistent with purifying selection acting on the coding regions. At the species level, the ratio *π*_*N*_*/π*_*S*_ ranged from 0.04 in Dall's and spectacled to 0.10 in finless porpoises (Table S3). The vaquita displayed an intermediary value of 0.06. Within each species, *π*_*N*_*/π*_*S*_ also varied markedly across lineages: in the harbor porpoise, *π*_*N*_*/π*_*S*_ ratios ranged from 0 in the North Pacific NP2 lineage to 0.21 in the Black Sea BS lineage; in the finless porpoises from 0.14 in EAF to 0.17 in YFP; 0.05 in the DP2 Dall's porpoise lineage; and 0.06 in SP2 spectacled porpoise lineage (Table S3).

The branch-specific non-synonymous to synonymous substitution rates (*d*_*N*_*/d*_*S*_, Fig. S3a) were fairly conserved across the phylogenetic tree and ranged from 0 in the finless porpoise to 1.08 in the harbor porpoise, with a median value at 0.12. A *d*_*N*_*/d*_*S*_ lower than one implies purifying selection. Thus, similar to *π*_*N*_*/π*_*S*_, the branch-specific *d*_*N*_*/d*_*S*_ suggested that the porpoise mitogenomes were mostly influenced by purifying selection. Furthermore, the *d*_*N*_*/d*_*S*_ ratios did not differ significantly among species (PERMANOVA, *p*-value = 0.49). Interestingly, the *d*_*N*_*/d*_*S*_ ratio was negatively correlated with the nucleotide diversity (Fig. S3b; Pearson's *r* = − 0.64, *p*-value = 0.01) suggesting that purifying selection removes deleterious mutations more effectively in genetically more diversified lineages.

**Figure 4:**
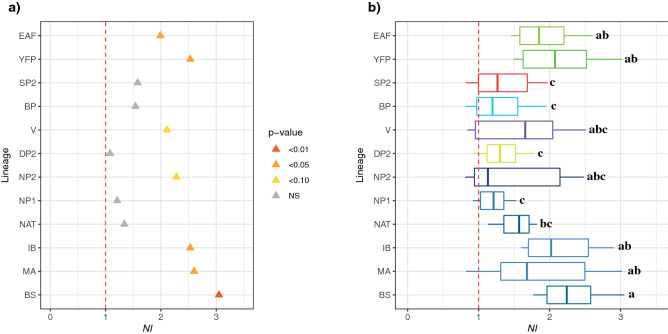
Mcdonald–Kreitman tests on the 13 protein coding regions of the mitogenome among porpoise subgroups. (**a**) Neutrality index (*NI*) estimated using the orca as outgroup. (**b**) NI distributions per mitochondrial lineages, calculated using various outgroups, including orca and all possible interspecific comparisons. The letters on the right of the boxplots indicate significant differences in the mean *NI* between the different lineages (i.e. boxplot with different letters are statistically different from one another). The red dashed lines represent the limit at which *NI* reflects positive (*NI* < 1), neutral (*NI* = 1), or purifying (*NI* > 1) selection. *NS* not significant. The acronyms are provided in Fig. 1.

The Mcdonald–Kreitman (MK) tests using first the orca as an outgroup showed that all the lineages for each species had neutrality index (*NI)* values greater or equal to one (Fig. 4a). In particular, some lineages displayed *NI* values significantly higher than one (G-tests, *p*-value < 0.05), consistent with a signal of purifying selection. These included the EAF and YFP lineages in the finless porpoises and the MA, IB and BS in the harbor porpoises (Fig. 4a). Vaquitas and NP2 harbor porpoises also displayed marginally significant *NI* values (*NI* > 2, *p*-value ≤ 0.10; Fig. 4a). The remaining lineages showed *NI*s close to one suggestive of selective neutrality. The MK tests applied to all pairs of interspecific lineages showed *NI* values often higher than one (Fig. 4b and Fig. S4a). The values were especially high (Fig. S4a) and significant (Fig. S4b) when comparing the harbor porpoise lineages (MA, IB, and BS) with the finless porpoise lineages (YFP and EAF). The variation in the distribution of *NI* among interspecific lineages (Fig. 4b) showed that these same lineages displayed significantly larger *NI* values compared to spectacled SP2 and Dall's DP2 porpoise lineages (PERMANOVA, *p*-value < 0.001 and all pairwise comparisons have a *p*-value < 0.04 after False Rate discovery adjustment). We observed a significant negative correlation between *π* and *NI* (Pearson's *r* = − 0.28, *p*-value = 0.003), suggesting that purifying selection could be stronger in lineages with small *Ne* or that demographic events impacted the polymorphism of these lineages.

### Demographic history

The vaquita displayed significant departure from neutral constant population size expectations with significant negative values for Fu and Li’s D* and F*, and Tajima’s *D*, even if this latter statistic was not significantly different from zero (Table 3). This result indicates a significant excess of singleton mutations compared to a neutral expectation, consistent with a bottleneck or a selective sweep. In contrast, the harbor porpoise NP1 lineage did not show any such significant deviation, even though all the statistics displayed negative values.

**Table 3:** Neutrality tests based on the site frequency spectrum.

Lineage	*D*	D*	*F*
Vaquita *(n* = *12)*	− 1.44^NS^	− 1.89*	− 1.84*
North Pacific 1 *(n* = *10)*	− 1.18^NS^	− 1.27^NS^	− 1.28^NS^

The neutrality tests were only applied to lineages where at the sample size (*n)* was at least 10. The statistics include the Tajima’s *D*, Fu and Li’s D*, and Fu and Li’s *F.*

*NS* Not significant.

*p-value < 0.05.

**Figure 5:**
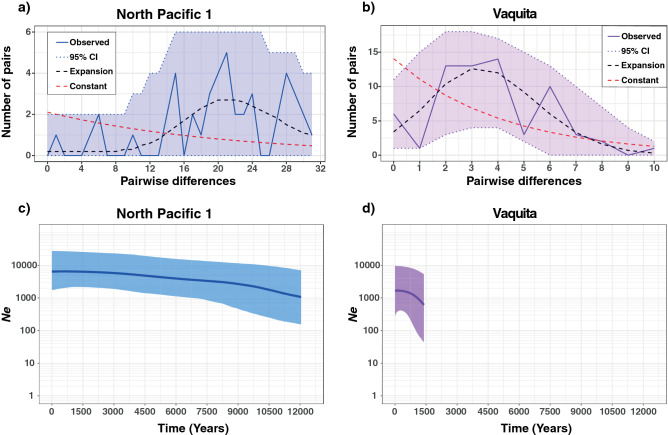
Mismatch distributions (**a**, **b**) and Bayesian Skyline Plots (BSPs) (**c**, **d**) for the North Pacific harbor porpoise 1 (NP1) lineage and the vaquita. The BSPs (**c**, **d**) show the temporal changes in mitochondrial diversity. The y-axis of the BSPs shows the genetic diversity expressed as the effective female population size (*Ne*). The bold line inside each BSPs represents the median value and thin lines the 95% HPD intervals. For both BSPs, the null hypothesis of a constant population size was rejected with a high confidence (Bayes Factors > 6).

The mismatch distribution and the coalescent-based BSP also captured this contrast (Fig. 5). For the North Pacific harbor porpoise NP1 lineage, the mismatch distribution was consistent with an ancient population expansion (Fig. 5a) with a modal value close to 20 differences on average between pairs of sequences. Despite the ragged distribution and large 95% CI, the best fitted model suggested an ancient increase in genetic diversity (*θ* = *2*·*Ne*·*µ*) by a factor of 40 after a period (*τ* = *2*·*t*·*µ)* of 18 units of mutational time. This old expansion was also detected by the BSP analysis (Fig. 5c). Indeed, NP1 displayed an old steady increase in *Ne* with time since the most recent common ancestor (*T*_*MRCA*_) 16,166 years before present (years BP) (95% CI 12,080–20,743), with a median *Ne* increasing from 1,660 to 6,436 (Fig. 5c). This model was strongly supported over the null hypothesis of a constant population size with all Bayes factors greater than 6.4. For the vaquita lineage, the mismatch distribution and the BSP analyses both supported a much more recent expansion than in NP1. The mismatch distribution (Fig. 5b) showed an increase of *θ* by a factor of 1,000 after a* τ* of four units of mutational time. The mode of the bell shape distribution for the best fitted model was around three differences among pairs of sequences, which is consistent with a recent population expansion. The BSP analysis (Fig. 5d) captured this expansion and explained the data significantly better than a constant population size model (Bayes factors > 8.7). This expansion was dated back to 3,079 years BP (95% CI 1,409–5,225), with median *Ne* increasing from 613 (95% CI 45–5,319) to 1,665 (95% CI 276–9,611). Thus, the estimated current *Ne* was 3–6 times lower than in NP1 (Fig. 5d).

## Discussion

The phylogeny of the Phocoenidae has been debated for decades, in part due to the lack of polymorphism and statistical power that came from the analyses of short fragments of the mitochondrial genome^17,42^. Using massive parallel sequences technologies, the analyses of newly sequenced and assembled whole mitogenomes from all the species and sub-species of porpoises provide a robust comprehensive picture of the evolutionary history of the porpoises. The phylogenetic relationships estimated here delivered a fully resolved evolutionary tree (Fig. 1b and Fig. S1). While most of the phylogenetic relationships were suggested previously^17,42^, the resolution and statistical support recovered here was maximal. Our results support the monophyly and branching of each species and sub-species. Moreover, the comparative view of the mitochondrial polymorphism within and among species provides one of the first attempts to bridge macro- to micro-evolutionary processes in a cetacean group (see also Ref.^84^). This perspective across evolutionary time-scales can shed light on the isolation dynamics and their drivers across the speciation continuum of the Phocoenidae.

### New insights into the biogeography of the Phocoenidae

The biogeographical history of cetacean species has been hypothesized to results of a succession of vicariant and dispersal events influenced by geological, oceanic, and climatic reorganization during the Late Miocene, Pliocene and early Pleistocene^1,17^. Changes in climate, ocean structure, circulation, and marine productivity opened new ecological niches, enhanced individual dispersal and isolation, and fostered specialization to different food resources. All these factors promoted the adaptive radiation in cetaceans which led to the extant species diversity in the odontocete families^85^. For example, the Monodontidae and Delphinidae are the closest relatives to the Phocenidae. They originated during the Miocene and displayed an accelerated evolution marked with the succession of speciation events during 3 Myr, leading to the extant species diversity in these groups^1,17^. The time calibrated phylogeny of the Phocoenidae (Fig. 2) suggests that porpoises also diversified following similar processes during the late Miocene until the early-Pleistocene (between 6 and 2 Myr). This timing is about 2–3 Myr more recent than those proposed by McGowen et al*.*^17^ and Steeman et al*.*^1^. It is worth mentioning that recent estimates proposed by McGowen et al*.*^86^ included four of the six porpoise species and were more in line with our estimates. The increase of the genetic information, node calibrations and number of sequences per species are known to influence phylogenetic inferences and divergence time estimates^6,87,88^. The use of complete mitogenomes, two node calibrations (instead of one), and several sequences per species in our study likely explain the difference compared to previous studies.

Consistent with previous findings^17,41,42^, the finless porpoises were the first species to split among the Phocoenidae. As the vast majority of the porpoise fossils found so far come from tropical or subtropical regions^41^, and considering their current predominant affinity for warm waters, the finless porpoises seem to be the last members of a group of porpoise species that adapted primarily to tropical waters. Interestingly, finless porpoises further diversified and colonized more temperate waters of the Yellow Sea and Sea of Japan (Fig. 1a). The five remaining porpoise species diverged ~ 4.0 Myr ago and all but the vaquita occupy temperate regions with an antitropical distribution (Fig. 1a). Harbor and Dall’s porpoises inhabit the cold water of the Northern hemisphere whereas spectacled and Burmeister’s porpoises are found in the Southern hemisphere. This result is consistent with the hypothesis that antitropically distributed cetaceans have evolved with the deep environmental changes that occurred during the late Pliocene and as a response to the fluctuations in surface water temperatures in the tropics, concomitant with the changes in oceanographic currents, marine productivity, and feeding areas^1,12,15^. About 3.2 Myr ago, the formation of the Panama Isthmus altered the tropical currents and water temperatures in coastal regions of the Pacific, and throughout the world oceans. It promoted the dispersion of numerous taxa from the Northern to the Southern Hemisphere^89^. This process is also a plausible driver that led to the antitropical distribution of the modern porpoises^41^.

During the porpoises' evolutionary history, a symmetric evolution took place approximatively at the same time in both hemispheres resulting in analogous ecological adaptations. In the Northern hemisphere, the split between Dall’s and harbor porpoises ~ 3.1 Myr ago led to offshore versus coastal specialized species, respectively. This pattern was mirrored in the Southern hemisphere with the split between the spectacled and Burmeister’s porpoises ~ 2.1 Myr ago which led also to the divergence between offshore versus coastal specialized species. Such a symmetric habitat specialization likely reflects similar ecological opportunities that opened in both hemispheres and triggered a convergent adaptation in the porpoises. Interestingly, this possible parallel offshore evolution observed in Dall’s and spectacled porpoises would have been accompanied by a convergent highly contrasted countershading coloration pattern with a white ventral side and black dorsal back side in both species. This color pattern is thought to be an adaptation to the offshore environment serving as camouflage for prey and predators^90^.

Resources and diet specializations are known to be a major driver of cetacean evolution as their radiation is linked to the colonization of new vacant ecological niches in response to past changes^85^. As small endothermic predators with elevated energetic needs associated with their cold habitat and small size, limited energy storage capacity and a rapid reproductive cycle, porpoises are known for their strong dependency on food availability^91,92^. These characteristics reinforce the hypothesis that their adaptive radiation has been strongly shaped by historical variation in food resources and should also be visible at the intraspecific level.

### Porpoises phylogeography and microevolutionary processes

The processes shaping the evolution of porpoises at the macro-evolutionary time scale find their origins at the intraspecific level (micro-evolutionary scale), with the split of multiple lineages within species that may or may not evolve into fully distinct and reproductively isolated species. We showed that all lineages forming the intraspecific subdivisions (sub-species or ecotypes) each formed a monophyletic group. All these distinct lineages found their most recent common ancestor within the last million years. These results corroborate previous phylogeographic studies suggesting that intraspecific subdivisions observed in many porpoise species were mediated by environmental changes during the last glacial cycles of the Quaternary^19,25,26,30^. The Last Glacial Maximum (LGM) and the subsequent ice retreat have profoundly shaped the phylogeographic patterns of many organisms, leaving behind multiple divergent lineages in many cetacean taxa that are vestiges of past environmental variations^2,6,9,10,25,27,30,93^. Adaptive evolution to different niches in response to past changes associated with variation in marine sea ice, primary production, and redistribution of feeding areas led to intraspecific divergence in many cetaceans in terms of genetics, behavior, morphology, and geographical distribution. For example, the specialization between coastal versus offshore ecotypes in bottlenose dolphins has been dated back to the LGM, and explains the observed patterns of genetic and morphological differentiation^9^. Likewise, the behavior, size, color patterns and genetic divergence among some different types of killer whales were attributed to specialization onto different food resources since the LGM^10^. The present study shows that analogous processes occurred in each porpoise species too.

The finless porpoises represent probably one of the best documented cases of incipient speciation related to habitat specializations among the porpoises. Consistant with the results of Zhou et al*.*^19^ based on whole genome sequencing, our mitogenome results dated the radiation of the finless porpoise species within the last ~ 0.5 Myr. This coincides with the profound environmental changes associated with the Quaternary glaciations. In particular, our results are congruent with the hypothesis suggesting that the diversification of the finless porpoises has been driven by the elimination of the Taiwan Strait associated with the sea-level drop during glacial periods^94^. Indeed, at least three land bridges connected Taiwan to mainland China since the last 0.5 Myr and could have enhanced the separation between the Indo-Pacific and the narrow ridged finless porpoises^94^. Likewise, we dated the emergence of the Yangtze finless porpoise to the last ~ 0.1 Myr, which is consistent with previous studies suggesting that the last glacial event have strongly determined the evolutionary history of this species^19,25,95^.

Similar to the finless porpoises, the harbor porpoises are also divided into several lineages previously recognized as distinct sub-species. The deepest split is observed between the North Pacific (*P. p. vomerina*) and North Atlantic lineages, and is deeper than the genetic divergence observed between the two species of finless porpoises. The lack of shared haplotypes between Pacific and Atlantic porpoises confirm previous results supporting their total isolation^96^. Their splitting time was estimated at ~ 0.86 Myr, which is consistent with the presumed time when the North Pacific porpoises colonized the North Atlantic basin^2^. The two ocean basins were last in contact across the Arctic approximately 0.7–0.9 Myr. ago, with estimated sea surface temperatures of *ca.* 0.5 °C^97^, which corresponds to the lowest temperature at which harbor porpoises are currently observed. Within the North Atlantic, the three known sub-species^2,30^ (i.e. *P. p. phocoena*; *P. p. meridionalis* and *P. p. relicta*) were also detected as distinct monophyletic groups based on the mitogenome (Fig. 1b and Fig. S1). Their evolutionary history has been strongly influenced by recent environmental changes during the Quaternary period^2^ and particularly the LGM^8,30,60^. For example, Tolley and Rosel^2^ discussed in great detail how major climatic shifts during the Quaternary constantly reshaped the distribution of harbor porpoise in the North Atlantic and the Black Sea (through episodes of isolation, colonization, contraction and expansion of different habitats) leading to the current divergence among sub-species (see also^30^). North Pacific horbor porpoises also showed cryptic subdivisions (i.e. NP1 and NP2 in Fig. 1b and Fig. S1). Although several studies observed genetic structure among Pacific porpoises^4,98^, none captured the deep divergence highlighted here. NP1 and NP2 displayed a level of divergence deeper than the one observed between the Iberian and Mauritanian harbor porpoises or between the Yangtze and East Asian finless porpoises. These two clades (NP1 and NP2) may also represent lineages that split during the LGM, with NP1 showing a steady increase in genetic diversity since the end of the LGM period 12 kyrs ago (Fig. 5c). These results are consistent with those of Taguchi et al.^4^ suggesting that climatic fluctuation during the Pleistocene shaped the genetic structure of Pacific harbor porpoises.

Compared to the finless and harbor porpoises, little is known about the Dall's, Burmeister's and spectacled porpoises. This is in part due to the limited number of observations and access to biological data (but see Refs.^26,27,99^), especially for the spectacled porpoise. Despite these limitations, our study revealed that the patterns and processes described for the finless porpoise and harbor porpoise may apply also to the majority of the other porpoise species. Previous studies identified multiple intraspecific subdivisions within the Dall's^26^ and Burmeister's^27^ porpoises. The long branches in the phylogenetic tree (Fig. 1b) for Dall's porpoise (DP1/DP2 lineages) and spectacled porpoises (SP1/SP2) imply that distinct evolutionary units may also exist in these species. Furthermore, their vast distribution (Fig. 1a) and divergence times among lineages (Tables S5) suggest that the different lineages in these species also split during the Quaternary glaciations. This is congruent with the study of Hayano et al*.*^26^ indicating that different lineages of Dall's porpoises from the west Pacific diverged in response to multiple events of population isolation occurring between 40,000 and 10,000 years ago during the LGM.

The vaquita contrasts strikingly with the other species with its narrow distribution, the smallest of all marine mammals (see Fig. 1a and Ref.^100^). Previous studies based on a short fragment of the mitochondrial *D-loop* and *Cyt-b* identified the Burmeister's porpoise as the closest relative to the vaquitas. However, the phylogenetic results reported here using the whole mitogenome support that the vaquita coalesce with the ancestor of the Burmeister's and spectacled porpoise with maximal support. The estimated split time of ~ 2.4 Myr ago between vaquita and the southern porpoises is consistent with the onset of the Quaternary glaciations, 2.6 Myr ago^101^. The fact that the vaquita is found in the northern hemisphere, while the Burmeister's and spectacled porpoises are in the southern hemisphere implies that some ancestors with cold-affinities from the southern species crossed the equator in the past and became isolated long enough to become the ancestors of the extant vaquita. The most parsimonious hypothesis is that the decrease in water temperature associated with a glacial maximum likely allowed vaquita’s ancestor to cross the equator and disperse to the Northern Hemisphere^102^. The current vaquita representatives thus form a relic population of the temperate southern species’ ancestor that crossed the intertropical zone. In contrast with previous mitochondrial studies that found no variation at the *D-loop*^103^, we observed 16 sites segregating along the entire mitogenome. Among them, 13 were singleton mutations. This extremely low nucleotide diversity was the lowest of all porpoise lineages, as illustrated by the extremely short branches in the phylogenetic tree (Figs. 1b and 2). The origin of the present mitochondrial diversity is also relatively recent, with a *T*_*MRCA*_ estimated at ~ 20,000 to 70,000 years with the phylogenetic approach and ~ 1,500 to 5,000 years with coalescent approach. The main reason behind this discrepancy is called “time dependency of molecular rates”^6,87^. Population genetics coalescent-based estimates reflect the spontaneous mutation rates, whereas phylogeny-based estimates reflect the substitution rates (i.e. mutations fixed among taxa). Contrasting with these recent estimates, the branch connecting to the ancestor of vaquitas and southern species dates back to ~ 2.4 Myr. This suggests that either additional lineages may have existed in the past and went extinct or that only a single lineage crossed the intertropical zone. Whole genome analyses may help enlightening the evolutionary history of this peculiar species.

### Genetic diversity and conservation

Maintenance of genetic diversity has been considered as an important factor in conservation biology. Genetic factors can contribute to the “extinction vortex” by a mutual reinforcement with demographic processes speeding-up population decline and increasing their extinction risk^104^. As a consequence, ideal conservation measures should be designed to maximize genetic diversity, especially through the management of evolutionary significant units (ESU)^105^. However, conservation status does not explicitly take this parameter into account since the relationship between IUCN status and genetic diversity is not always straightforward^106^. In this study, the genetic diversity of each porpoise species correlates well with its IUCN status, especially when we account for intraspecific subdivision. The *Critically Endangered* taxa, such as the vaquitas or the YPF finless porpoises displayed extremely low *π* suggesting a low *Ne*. The *Endangered* (EAF finless porpoise) and *Vulnerable* (Black Sea harbor porpoise) taxa displayed low to average *π*. *Least Concern* taxa (e.g. North Atlantic harbor porpoise and Dall’s porpoise DP2) exhibited higher *π*, suggesting larger *Ne* and/or the presence of internal subdivision. This link between *π* and the IUCN conservation status may thus provide a useful proxy to assess the conservation status of taxa for which an IUCN status has not been yet established, due to data deficiency. For example, the Iberian harbor porpoise population is among the marine mammals displaying the highest stranding and by-catch rates reported^37^. The low genetic diversity reported in the present study represents thus an additional signal indicating how possibly vulnerable are these Iberian porpoises. On the other hand, spectacled porpoises represent currently one of the least known cetacean species. Their genetic diversity is comparable to the one observed in the Dall's porpoise DP2 lineage or the North Atlantic (NAT) harbor porpoise lineage, suggesting these populations display large *Ne*.

Mitochondrial diversity may not always be a good proxy of population abundance. Other evolutionary processes than just demography may impact genetic diversity^107^ (i.e., such as natural selection). The *d*_*N*_/*d*_*S*_ and *π*_*N*_*/π*_*S*_ lower than 1 highlighted in this study would be usually indicative of purifying selection acting on the mitochondrial genetic diversity. The negative relationships observed between *π* and *d*_*N*_/*d*_*S*_ (Fig. S3b) lend further support to this hypothesis, suggesting that purifying selection is more effective in large populations as predicted by the neutral theory^68,69^. Surprisingly, the MK-tests suggested that purifying selection was prevailing on the mitochondrial genomes of the endangered porpoises with *NI* values larger than 1. In contrast, selective neutrality could not be rejected for less threatened species. At first glance, this result for the endangered porpoises seems counter intuitive. Purifying selection is expected to be less effective in lineages where population size is very small*,* since genetic drift is expected to outperform selective forces^67^. However, when a lineage harbors a low *Ne*, slightly deleterious variants are expected to increase in frequency and segregate for a longer time without being fixed. Parsch et al*.*^71^ showed in different populations of *Drosophila melanogaster* that this effect will increase the number of polymorphic nonsynonymous mutations compared to the divergent nonsynonymous mutations (*π*_*N*_ ≫ *d*_*N*_). Hence, despite the reported increase of *d*_*N*_/*d*_*S*_ in endangered taxa, it induces a bias in the neutrality index toward positive values creating an artefactual signal of purifying selection. The higher values of *π*_*N*_*/π*_*S*_ observed in the endangered porpoise taxa (Table S3) and the negative relationships reported between *π* and *NI* support this hypothesis. All these elements suggest that demographic processes rather than selective forces drive the genetic diversity of the mitochondrial genome, and lead to high values of NI or *d*_*N*_/*d*_*S*_ in the endangered taxa.

## Conclusions

Using complete mitochondrial genomes, we reconstructed a comprehensive picture of the evolutionary history of the Phocoenidae. Besides clarifying the debated phylogenetic relationships among porpoises, our results provided new insights into the process driving species diversification in the porpoises across the speciation continuum. Similar to the Delphinidae, the Phocoenidae recently radiated in response to past environmental variation, adapting to different environments, ecological niches, and food resources. Furthermore, our results suggested that the processes governing their divergence at the macro-evolutionary scale find their origins at the micro-evolutionary scale. We revealed cryptic genetic subdivision for several taxa suggesting that our knowledge about many species, especially the data deficient southern species, is still scarce. Finally, the level of mitochondrial genetic diversity within a species seems to be primarily driven by demographic processes, rather than natural selection and turned out to be a good proxy for the conservation issues reported in these groups (i.e. Yangtze finless porpoises or vaquita).

The phylogenetic inferences in this study rely only on the mitogenome. A single genetic marker may not be fully representative of the species evolutionary history because individual gene trees may sometimes differ from the species tree^108^. Selection, incomplete lineage sorting and introgression can create discordance between gene trees and the species tree^108^. This issue is expected to be especially problematic in closely related species where introgression can still occur^109^, in group of species that rapidly radiated^84,110^, or in species with large effective population sizes where genetic drift may be inefficient to sort out lineages among groups and where selection can have a significant impact^109,111^. The macroevolutionary timescale of divergence among porpoise species associated with their small population size, and their largely allopatric current distributions, imply that ILS, introgression, and selection should have a limited impact. We are thus confident in the phylogenetic inferences made from the mitogenome data. Nevertheless, it will be important in future studies to use genome scale data to validate and complement the phylogenetic inferences of the present study and provide finer resolution of the evolutionary processes within and among species.

## Supplementary information

Supplementary Information.

## Data Availability

Mitochondrial genome assemblies and sequence reads were deposited on NCBI under the BioProject ID: PRJNA659918. Genbank accession numbers of mitochondrial sequence assemblies referring to individual specimens are listed in table S2. Alignment data and scripts are available via  the IRD Porpoises genetics and genomics Dataverse (10.23708/QBIUMI).
